# A Polarizable Forcefields for Glyoxal Acetals as Electrolyte Components for Lithium‐Ion Batteries

**DOI:** 10.1002/open.202400134

**Published:** 2024-07-31

**Authors:** Adriano Pierini, Vanessa Piacentini, Juan Luis Gómez‐Urbano, Andrea Balducci, Sergio Brutti, Enrico Bodo

**Affiliations:** ^1^ Department of Chemistry University of Rome La Sapienza P. Aldo Moro 5 00185 Rome Italy; ^2^ Institute for Technical Chemistry and Environmental Chemistry Friedrich-Schiller University Jena Philosophenweg 7a 07743 Jena Germany; ^3^ Center for Energy and Environmental Chemistry Jena (CEEC) Friedrich-Schiller University Jena Philosophenweg 7a 07743 Jena Germany; ^4^ Istituto dei Sistemi Complessi Consiglio Nazionale delle Ricerche P. Aldo Moro 5 00185 Rome Italy

**Keywords:** Polarizable Molecular Dynamics, Electrolytes, Force Field, Glyoxal Diacetals

## Abstract

In this work we have derived the parameters of an AMOEBA‐like polarizable forcefield for electrolytes based on tetramethoxy and tetraethoxy‐glyoxal acetals, and propylene carbonate. The resulting forcefield has been validated using both ab‐initio data and the experimental properties of the fluids. Using molecular dynamics simulations, we have investigated the structural features and the solvation properties of both the neat liquids and of the corresponding 1 M LiTFSI electrolytes at the molecular level. We present a detailed analysis of the Li ion solvation shells, of their structure and highlight the different behavior of the solvents in terms of their molecular structure and coordinating features.

## Graphical abstract

Molecular dynamics simulations of electrolytes based on glyoxal acetals reveal the different solvation patterns of the Li^+^ ion and how they depend on the structure and interplay of solvent molecules.

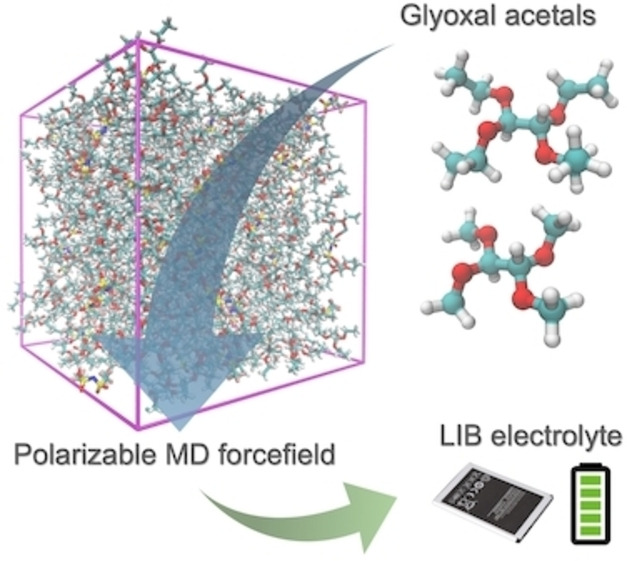

## 1 Introduction

The electrolyte formulation plays a critical role in determining the voltage and capacity attainable by rechargeable lithium‐ion batteries (LIBs), as well as their safety specifications and operative conditions. In state‐of‐art commercial LIBs, the electrolyte is constituted of a conductive lithium salt dissolved in mixture of liquid organic carbonates.[^1^, ^2^] The standard in this class of electrolytes is the so‐called LP30, a 1 M LiPF_6_ solution in a 1 : 1 mixture of ethylene carbonate and dimethyl carbonate.

Although its physico‐chemical properties have been a great propeller for the success of the LIB technology, the current trends in the industry of green energy expose the actual limitations of LP30 and of other carbonate‐based electrolytes in terms of stability, safety and materials toxicity.[^3^, ^4^, ^5^, ^6^, ^7^]

Moreover, the development of next‐generation lithium batteries imperatively requires beyond the‐state‐of‐the‐art electrolytes. For example, the conventional LP30 electrolyte shows incompatibility issues with many high‐capacity anode materials like Si and Si/Gr, due to the large volumetric changes occurring upon lithiation/delithiation of the electrodes (the so called “breathing behavior”).^[8]^ The inability of LP30 to form a stable solid‐electrolyte interphase (SEI)[^9^, ^10^] under these conditions, forces the use of large amounts of sacrificial electrolyte additives.^[11]^

**Figure 1:**
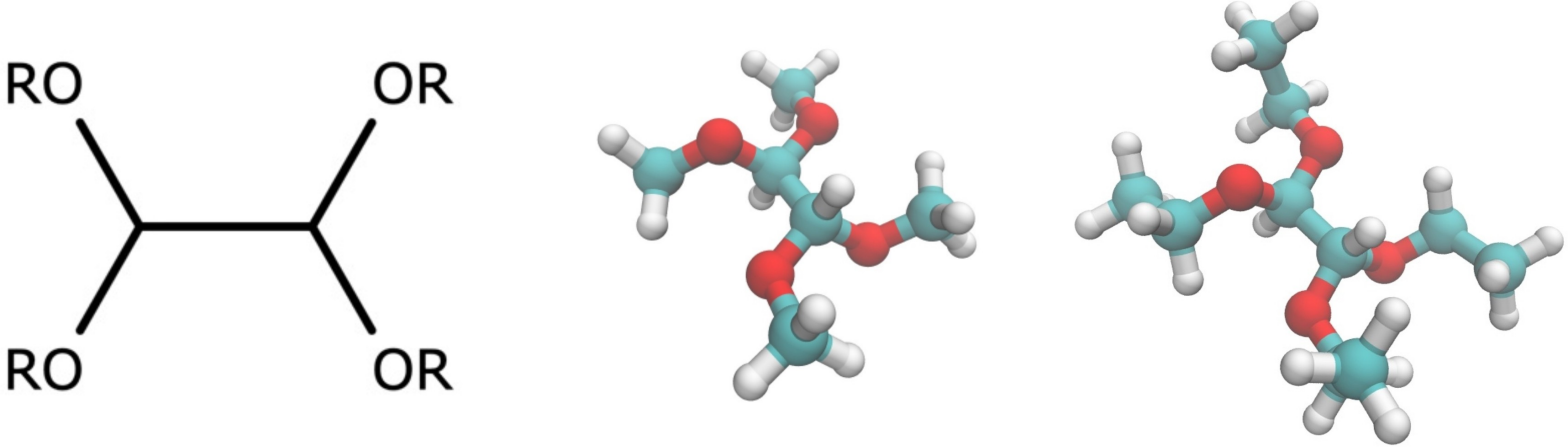
From left to right: general structure of glyoxal diacetals; 1,1,2,2‐tetramethoxyethane (TMG); 1,1,2,2‐tetraethoxyethane (TEG).

Among the vast field of possible candidates for replacing or improving the electrolytes based on carbonate solvents, a recent option is represented by compounds belonging to the class of glyoxal acetals,[^12^, ^13^, ^14^, ^15^, ^16^] such as 1,1,2,2‐tetramethoxyethane (also tetramethoxy‐glyoxal acetal, TMG), and 1,1,2,2‐tetraethoxyethane (also tetraethoxy‐glyoxal acetal, TEG). These materials are easily synthesized and commercially available, and their precursor (dialdehyde glyoxal) is widely available and inexpensive.^[17]^ Compared to most of the commonly organic carbonates, TMG and TEG display low toxicity, relatively high flash point and low vapor pressure, all properties that make them appealing candidates for improving the safety and thermal stability requirements of lithium batteries.[^12^, ^13^] Pure TMG and TEG have low relative permittivity (ϵ=3.5 and ϵ=2.5, respectively), but nevertheless, they can easily solubilize alkaline TFSI salts,^[12]^ thus making it possible to prepare electrolytes with molar concentration larger than 1 mol L^−1^ suitable for applications in energy storage systems. The molecular structures of TMG and TEG are shown in Figure 1.

Electrolytes based on 1 M LiTFSI in TMG or TEG, used in combination with graphite and carbon‐based electrodes, display good electrochemical stability and film‐forming ability,^[13]^ with the TEG‐based electrolyte exhibiting higher capacity and stability than TMG at both low and high C‐rates. At the same time, a clear shortcoming in glyoxal‐based electrolytes is represented by the coupling of low ionic conductivity and high viscosity.^[14]^ For this reason, binary mixtures of these solvents with a high‐permittivity one, like organic carbonates, have been suggested to increase conductivity.[^14^, ^15^, ^16^, ^18^] As expected, this addition allows to attain higher values in ionic conductivity, with a strong dependence on the mixture composition over the whole range of acetal‐to‐carbonate ratio.[^14^, ^15^] Köps et al.^[15]^ compared the binary mixtures of TEG and TMG with ethylene carbonate (EC), dimethyl carbonate (DMC) and propylene carbonate (PC) at different ratios, ultimately finding that mixing PC with TEG in a 7 : 3 weight ratio provided the best balance between improved thermal stability and transport properties, while still preventing significant co‐intercalation of the carbonate solvent. This 7 : 3 PC : TEG LiTFSI 1 M electrolyte formulation displays an ionic conductivity of 3.5 mS ⋅ cm^−1^ at 20 °C and, combined with carbon‐based electrodes, shows particularly promising performances for application in high temperature devices.[^15^, ^16^] More in detail, enhanced capacity values were delivered by LIBs assembled with this novel electrolyte while operating at temperatures of 40 and 60 °C. Despite further examinations about its performance at lower temperatures are needed, the extended liquid range and high thermal stability of PC:TEG LiTFSI 1 M electrolyte make it a suitable candidate for applications in which high safety and a wide range of temperature are required.

The film‐forming ability of the glyoxal acetals represents a strong appeal for the development of high‐capacity Si and Si/Gr electrodes. Gehrlein et al.^[19]^ reported that 1 M LiTFSI in pure TEG could form a uniform and flexible SEI on Si/Gr which maintains a stable coverage on the negative electrode during its large volume changes, ultimately resulting in high cell capacity (488 mAh ⋅ g^−1^ after 100 cycles). On the other hand, despite the overall similar morphology and composition of the SEI, the performances of TMG were poorer, with capacity fading after the first ten cycles. A similar ability of TEG/TMG to form a SEI which can effectively accommodate large volume expansions was also verified on Fe_2_O_3_/C‐based anodes delivering even 800 mAh g^−1^ at 60 °C.^[20]^ Moving outside the field of LIBs, several investigations have also reported about the possibility of employing glyoxal acetals as electrolyte components in different battery chemistries, like lithium‐sulfur,^[21]^ sodium‐ion^[22]^ and potassium‐ion batteries.[^23^, ^24^]

Despite the extensive electrochemical characterization carried out in these works, no investigation has been conducted so far (to the best of our knowledge), neither spectroscopically nor theoretically, about the relationship between solvation, local structure and transport mechanisms in glyoxal acetals at the molecular scale. In this work, we present the first molecular dynamics (MD) simulations of TMG/TEG systems, using an appositely developed polarizable forcefield (FF).

Compared to the fixed point‐charge approximation, polarizable FFs generally provide a better description of both structural and dynamical properties of condensed phase materials, especially in systems presenting a large amount of mobile charge carriers, like ionic liquids and liquid electrolytes.[^25^, ^26^, ^27^, ^28^, ^29^, ^30^] In the context of nonpolarizable FFs, a common practice is to scale down the atomic charges to account for electronic charge polarization in an effective, averaged way. Although this approach can be used to obtain a good fit with target experimental properties (like ionic conductivity or viscosity), it may come at the price of a possible loss of accuracy for what concerns structural and solvation properties and may lead to a loss of transferability.[^25^, ^31^, ^32^]

The effects of electronic polarization can be explicitly included in MD through different models.^[33]^ Here we have decided to adopt the inclusion of polarization through the explicit calculation of the induced atomic dipoles and of the ensuing multipolar expansion of the electrostatic energy. The resulting potential energy model is widely known as AMOEBA.^[34]^

The construction of this kind of force field requires the computation of accurate ab‐initio data on the isolated molecules that include electrostatic multipoles and polarizabilities and their fitting to atom‐centered expressions. In this work, we present newly computed parameters for TMG, TEG, TFSI^−^ and PC with the latter being a possible co‐solvent in binary mixtures. The FF validation was done using both ab‐initio data and measured bulk observables. We used the FF to perform MD simulations of the pure liquids and of their corresponding 1 M LiTFSI electrolytes to investigate the solvation properties at the molecular scale, to extract the structure of the solvation shells and ultimately to rationalize possible strategies for improving the electrolyte formulations.

## 2 Computational Methods

The molecular species TMG, TEG, PC and TFSI^−^ were parametrized according to the AMOEBA model,^[35]^ where the potential energy is given by the sum:

(1) $$U=U_{b}+U_{a}+U_{sb}+U_{o}+U_{t}+U_{vdw}+U_{el}^{p}+U_{el}^{i}$$

Where *U_b_, U_a_, U_sb_, U_o_* and *U_t_* are the typical intra‐molecular valence interactions (respectively, bond stretching, angle bending, stretch‐bend coupling, out‐of‐plane bending and torsional rotations) and the last three terms describe the van der Waals and electrostatic interactions from permanent multipoles *U* ^*p*^_*el*_ and induced dipoles *U* ^*i*^_*el*_.

The parametrization of the four species (TMG, TEG, PC, TFSI^−^) followed the procedure reported in the reference paper of Poltype2.^[36]^ In particular, the parameters for electrostatic multipoles were fitted on the electrostatic potential calculated from MP2/aug‐cc‐pVTZ electron densities. Atomic polarizabilities, van der Waals and valence parameters were directly transferred from database matching atomic types, while few missing torsional angles were fitted from potential energy scans at the ωB97X‐D3/6‐311+G*//ωB97X‐D3/6‐31G* level. The derived FF parameters are available in Section S2. Molecular dynamics with the newly developed FF was performed using the Tinker‐HP^[37]^ program. For each system, a cubic simulation box containing approximately 16,000 atoms was first relaxed in NPT ensemble (1 bar pressure and 293 K temperature), for 1 ns. Thermal equilibration at NVT (293 K) then followed for 5 ns. The statistical production in NVT conditions (293 K) was run for 6 ns for the electrolyte systems, while only for 2 ns for the pure solvents, times that were sufficient to achieve convergence of the computed quantities. Additional details of the simulations are reported in Table S1. The equations of motion were integrated with multi‐timestep propagator RESPA,^[38]^ using an inner timestep of 0.25 fs and an outer timestep of 2 fs (1 fs during NPT relaxation). The reproducibity of the structural properties of simulations employing 1 fs and 2 fs timesteps was verified for one of the systems (PC+LiTFSI 1 M). The Bussi thermostat and Berendsen barostat were used for controlling the thermodynamic conditions. The cutoffs for van der Waals interactions and real‐space Ewald summation were chosen to be 12 Å and 7 Å, respectively.

For the FF validation, the ab‐initio potential energy curves were calculated with the SAPT2+3 method^[39]^ implemented in the PSI4^[40]^ package, using an aug‐cc‐pVDZ basis set. The interaction energies of gas‐phase molecular clusters were instead computed with ORCA^[41]^ using the ωB97X‐D3 functional and def2‐TZVP basis, adding geometric counterpoise corrections (gCP) for basis set superposition errors.^[42]^

### 2.1 Validation of the Forcefield

In general, the agreement between the energy from the AMOEBA FF and the ab‐initio data is very good both in terms of intermolecular cohesive energy and of individual solvent‐ion and ion‐ion interactions.

**Figure 2:**
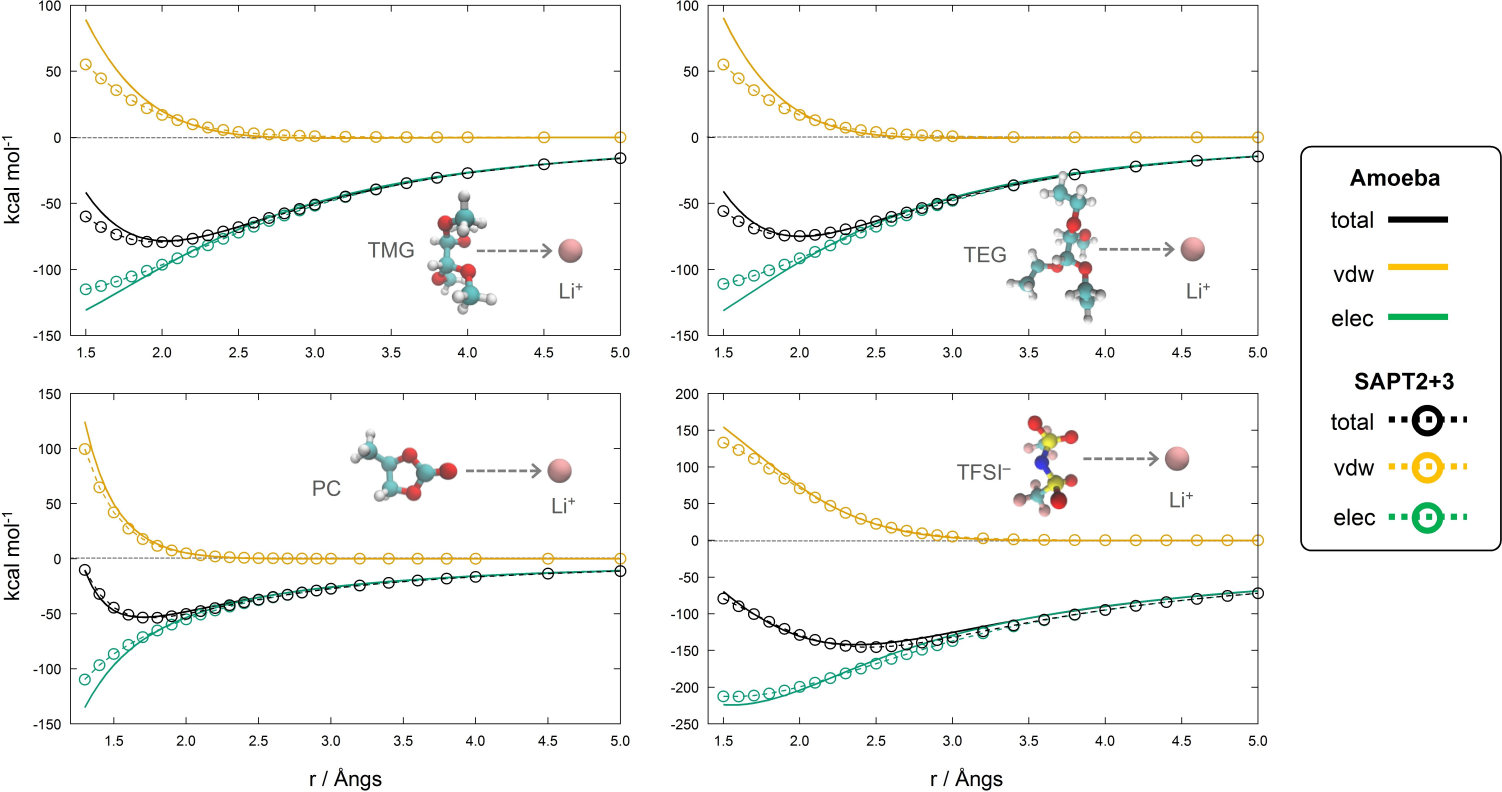
Interaction potential energy (kcal/mol) curves of a) TMG/Li^+^, b) TEG/Li^+^, c) PC/Li^+^, d) TFSI^−^/Li^+^. Intermolecular energy calculated with the FF (full lines) is compared with ab‐initio SAPT2+3 interaction energy (dashed lines). Total energy (black) is decomposed in electrostatic (green) and van der Waals (yellow) contributions. The intermolecular distance in a), b) d) is measured between Li^+^ and the solvent/anion center of mass, while in c) it is measured from the carbonyl oxygen of PC.

The results for the latter case are shown in Figure 2. We have compared the Li^+^‐solvent and Li^+^‐TFSI^−^ interaction energy calculated via the FF (lines) with the one stemming from ab‐initio calculations (circles) along an arbitrary cut of the potential energy surface obtained by gradually increasing the distance between Li^+^ and the molecular centers of mass (com). The molecular partner geometry was held fixed during this procedure to ensure that the FF and ab‐initio energies were computed at the same coordinates. The ab‐initio energy was decomposed into the SAPT contributions to allow a direct comparison, not only of the total energy (black lines), but also of the van der Waals interactions (yellow lines) and of the electrostatic ones (green lines) that include both static and induced contributions.

The Li^+^‐solvent (panels a, b and c) and Li^+^‐TFSI^−^ (panel d) potential energy curves display an almost perfect overlap at distances greater than the minimum, hence implying an excellent performance of the FF in reproducing the long‐range interactions. A small deviation between the FF energy and the ab‐initio one can be noted for distances below the energy minimum and is due to an overestimation of van der Waals repulsion in the FF that is however counterbalanced by an opposite sign error on the electrostatic. This effect is more notable for TEG and TMG where the relatively large conformational freedom of the branched molecule limits the accuracy of the parametrization at close interaction distance. It is worth noting that the calculation of the FF parameters has been carried out using one specific conformer, the minimum energy one.

**Figure 3:**
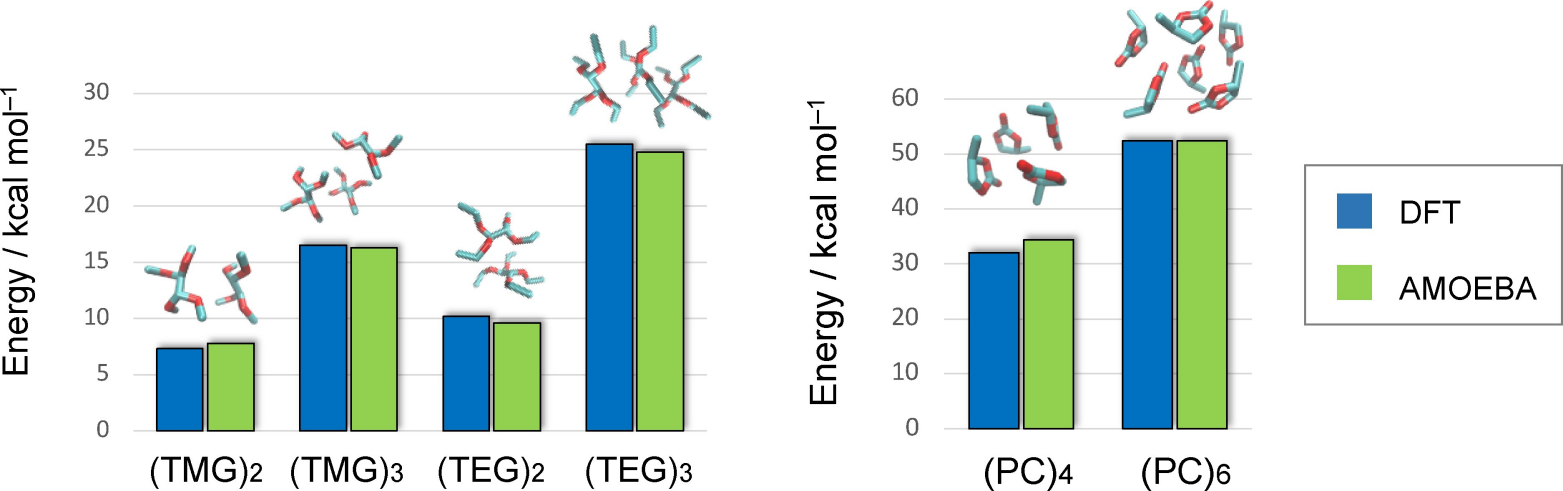
AMOEBA (green) and DFT (blue) interaction energy of small clusters of pure solvent molecules. The x axis labels indicate the chemical formulae of the (solvent)_n_ cluster where n is the number of molecules. The structures of the cluster for which the energy are reported is shown on top of the vertical bars.

The performance of our FF data in evaluating the cohesive energy of the fluid with respect to an ab‐initio reference is illustrated in Figure 3 where we have reported the interaction energy of small clusters of solvent molecules. The interaction energy is calculated as the difference between the total potential energy of the cluster and those of the monomers in their respective equilibrium geometry. The agreement is very satisfactory with the FF energy being consistently comparable to the DFT results. Even when considering larger aggregates (e. g. (PC)_6_), the FF interaction energy matches that of the high quality DFT calculations. This shows how, in such neutral molecular clusters, the many body expansion terms that build up the electrostatic energy converge quite rapidly, and the FF expansion remains accurate.

Overall, these results clearly show the accuracy of the AMOEBA approach in reproducing, not only the structural, but also the energetic properties of the molecular ensembles. This is even more remarkable when considering that the force field has been parametrized without the need of complicated adjustments of its parameters or extensive back‐fitting from experimental/ab‐initio data.

## 3 Results and Discussion

Our simulations include a first set of dynamics of the three neat solvents (TMG, TEG, PC) and their respective 1 M LiTFSI solutions (electrolytes). In addition, we have also simulated a 1 M solution of LiTFSI in a mixture of PC/TEG in a weight ratio 7 : 3 to evaluate the effect of the acetal as an additive to a common solvent (PC) that is often used in mixtures.

### 3.1 Neat Liquids

We begin by briefly presenting the data pertaining to the pure solvents, so that we have also the opportunity to present additional validation of the FF by comparing its predictions to bulk experimental data.

**Figure 4:**
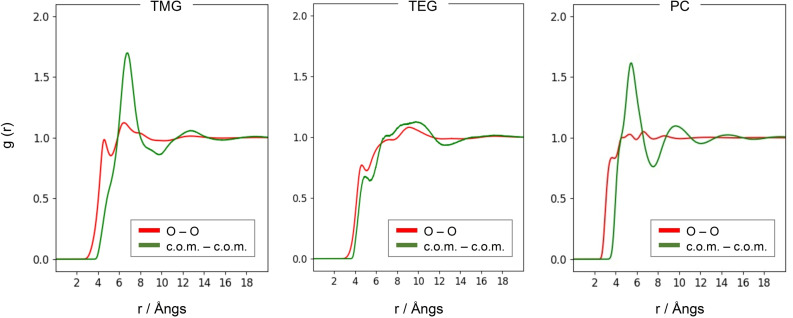
Radial distribution functions of the oxygen atoms and of the molecular centers of mass for the three neat solvents.

The structure of the three neat solvents is essentially determined by their molecular structure and, in particular, by whether the oxygen atoms are either exposed for mutual interaction or are hidden inside the alkyl chains. The difference in the structure between the three liquids can be outlined from the radial distribution functions (rdfs) reported in Figure 4. Two types of rdfs have been plotted: the first ones (red lines) are computed between all the oxygen atoms while the second (green lines) are between the molecular centers of mass.

Both neat PC and TMG show evidence of a structure at short‐range owing to the availability of the oxygen atoms in their molecular structures and, in the case of PC of its dipole moment. The rdfs in Figure 4 show that in PC the average first neighbor distance is around 5 Å while in TMG it is 7 Å, the latter having a larger molecular volume. Neat TEG, on the other hand, does not show any appreciable short‐range structure due to the prevalence of alkyl chains interactions that almost prevent any clustering between the molecules. Essentially, TEG appears as an unstructured liquid dominated by dispersive interactions.

Neat liquid bulk observables such as density and evaporation enthalpy are reported in Table 1. Our newly developed FF accurately predicts both, aligning well with existing experimental data.

**Table 1:** Computed (MD) vs experimental (expt.) densities (ρ) and evaporation enthalpies (▵H_ev_) of the pure solvents at 20 °C. The experimental data of ▵H_ev_ for TMG is available only for 93 °C.

Solvent	ρ (g/cm^3^)		▵H_ev_ (kcal/mol)	
	MD	exp.	MD	exp.
TMG	1.01	1.01^[a]^	13.8	10.25^[c]^
TEG	0.96	0.92^[a]^	17.1	–
PC	1.22	1.20^[b]^	15.5	17.1,^[d]^ 14.7^[e]^

^[a]^ data taken from supplier; ^[b]^ From ref. [43]; ^[c]^ from ref. [44] ^[d]^ from ref. [45]; ^[e]^ from ref. [46].

This, together with the results in the previous section, demonstrates that the new FF correctly evaluates both structural and energetic observables, spanning molecular and bulk levels. Hence, we expect that the results we are going to present on more complex systems (electrolytes) will be produced with a great degree of accuracy. In this context, the structural and ionic transport properties of the electrolytes will also be used as stringent tests of the quality of the force field as we will show in the section.

### 3.2 LiTFSI 1 M Solutions in Neat Liquids and Mixture

**Figure 5:**
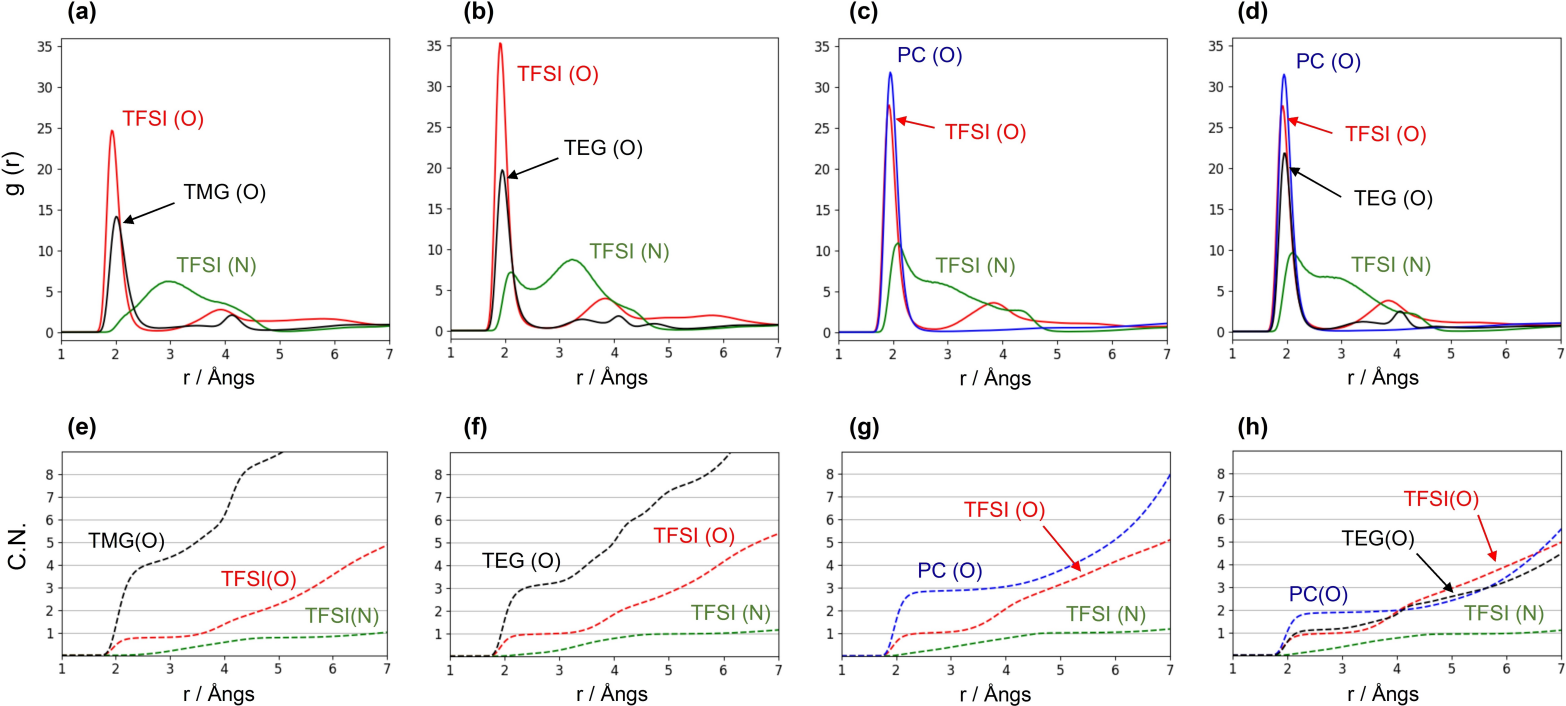
Li‐solvent and Li‐anion radial distribution functions (upper panels) and running coordination numbers (lower panels) for a) TMG+1 m LiTFSI, b) TEG+1 m LiTFSI, c) PC+1 m LiTFSI d) PC/TEG+1 m LiTFSI electrolytes.

Once demonstrated the neat solvent properties, we turn to the analysis of the geometric features of the local coordination shell of the Li cation in the 4 electrolytes. The relevant rdfs concerning Li‐solvent and Li‐TFSI distances are plotted in Figure 5. In the first set of panels from left to right we have the Li−O(solvent), the Li−O(TFSI) and the Li−N(TFSI) distances for TMG, TEG, PC and the PC:TEG mixture respectively. Obviously, this choice is inevitable since the Li coordinating atoms both in the solvents and in the anion are oxygens. In the second row of Figure 5, we have reported the volumetric running integrals of the rdfs that count the coordination numbers (atom‐wise). For both TMG and TEG, the average first neighbor coordinating distance for oxygen is ~1.8–2.2 Å with a prevalence of the shorter distance for TEG (figure 5, panels (a) and (b), black lines). The TFSI oxygens are localized at roughly the same distances (panels (a) and (b), red lines). However, the average coordination number of TMG oxygen in first shell (for distances less than 3 Å) is 4, while that of TEG is between 3 and 4. This is probably due to the bulkiness of the TEG molecule The coordination number of the TFSI oxygen is essentially one in both cases. This means that when TFSI is bound to Li^+^, it acts as a monodentate coordinating agent in both solvents where only one of its oxygen atoms is binding the cation. The TFSI nitrogen is generally considered a less coordinating site and its interaction is driven by a screened electrostatic interaction. In our case, in fact, it acts a larger distance with broad peaks at 3–3.5 Å (green lines) and, again, its integral shows that the inner coordinating shell of Li^+^ contains only one TFSI anion at most. A small difference between TMG and TEG can be spotted in the way in which TFSI^−^ is bound to the Li cation: for the latter, we can see a residual direct coordination of the amide nitrogen at short distances identified by the peak at 2.1 Å in the Li‐TFSI(N) rfd (figure 5, panel b, green line) that is completely absent in TMG. PC has a radically different molecular structure than TMG and TEG, but its solvation structure, resembles that of TEG. The average inner shell count for oxygen is 4 with one of the spots occupied by a TFSI one. The reduced size of PC with respect to TEG, allows the TFSI anion to reside near to the Li cation with an evident effect on the proximity of its nitrogen (green line, panel (c)).

When PC is mixed with TEG, they both share (on average) solvation of the Li cation. From panel (h) of Figure 5, we can see that, while one TFSI anion remains in the first shell, the remaining 3 spots are occupied by oxygens from PC and TEG molecules. Independently of the solvent, all O−Li coordinating distances remains localized within a rather narrow range between 1.8 and 2.2 Å.

**Figure 6:**
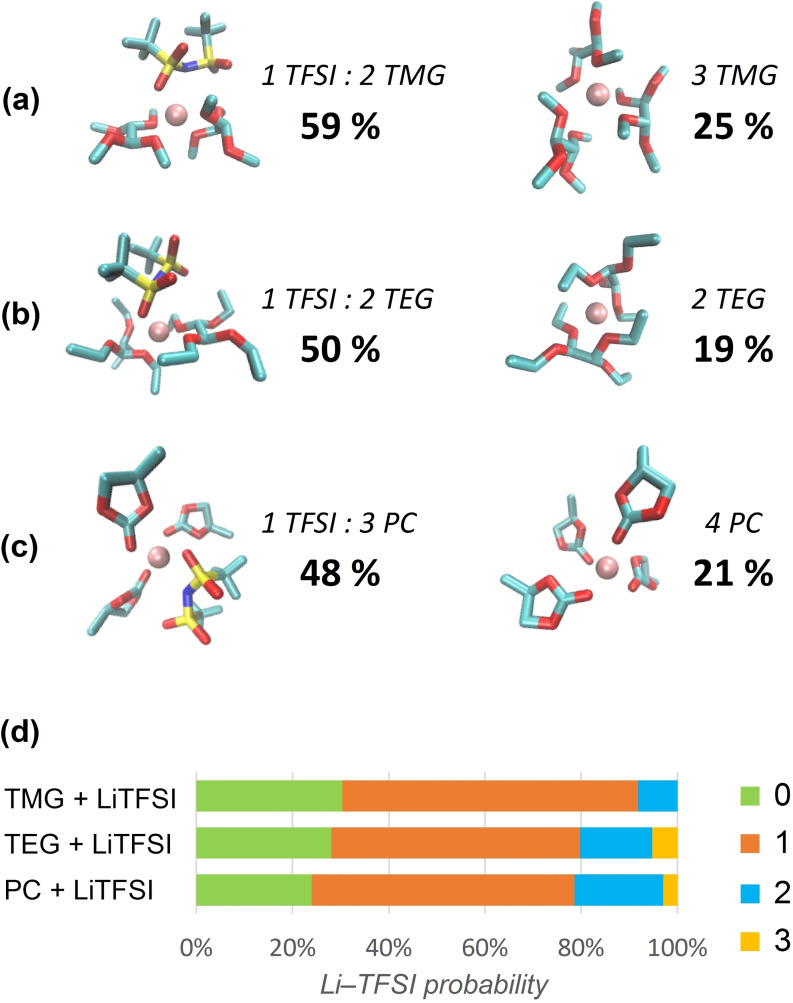
Most probable configurations of Li^+^ first solvation shell in a) TMG+LiTFSI, b) TEG+LiTFSI and c) PC+LiTFSI; and d) time‐averaged residence probability of TFSI^−^ anions inside the first solvation shell of Li^+^ for the same electrolytes.

A more refined analysis of the kind of structural arrangements in the immediate surroundings of the Li cation is displayed in Figure 6. In panels (a), (b) and (c) we report the two most likely structural organizations for TMG, TEG and PC respectively.

In the case of TMG (panel (a)), most of Li cations are either penta‐coordinated (~60 %) or hexa‐coordinated (25 %). The former configuration includes one TFSI^−^ (monodentate) and two TMG (bidentate) ligands. The latter, instead, is the most likely coordination of a dissociated Li cation and includes three bidentate TMG acetals.

In TEG, the situation is less clear, and a more disordered geometry emerges in the first solvation shell of the cation. The prevalent configuration (50 %) consists of a penta‐coordinated Li^+^ that uses 4 oxygen atoms from two TEG molecules and remains attached to an oxygen of a TFSI anion. In another probable configuration (~20 %) the Li cation is dissociated from TFSI and surrounded by only two TEG molecules with each using two oxygen atoms to provide a tetracoordinated environment.

PC solvation is dominated (~50 %) by a configuration where Li^+^ is tetra‐coordinated to a single oxygen of TFSI^−^ and where three PC carbonyl oxygen atoms partake to the solvation shell. Around 20 % of Li^+^ is dissociated from TFSI^−^ with a resulting tetracoordinated surrounding with 4 PC molecules.

In panel (d) of Figure 6, we report the averaged probability of counting 0 to 3 TFSI anions in the proximity of the Li cation. The green bars identify the probability of finding zero TFSI anions near Li^+^, i. e. they estimate the number of fully dissociated ionic couples. This number is more than 20 % in all 3 solvents with TMG the one where nearly 30 % of ionic couples are fully dissociated. This percentage decreases in TEG and even more in PC. The large majority of associated LiTFSI ionic couples, has a 1 : 1 configuration (orange bars) as shown also by the geometric analysis above. A sizable percentage of cations is coordinated to two TFSI anions. This happens only seldom in TMG (<10 %), is more frequent in TEG (~15 %) while in PC, it represents a substantial fraction of the population (~20 %). Surprisingly, in both TEG and PC a small and residual number of cations coordinate 3 anions.

**Figure 7:**
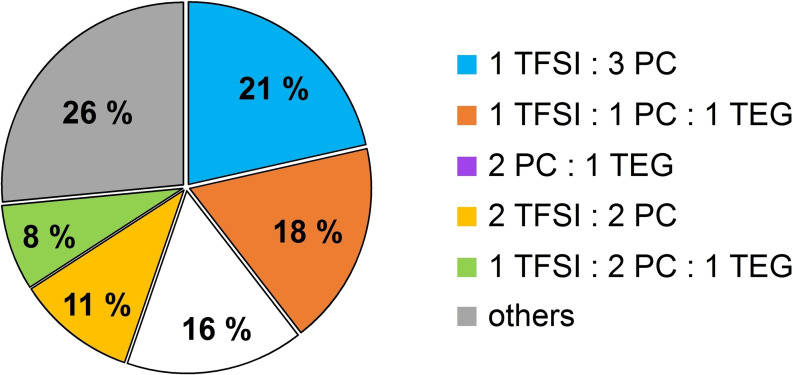
Average composition of Li^+^ first solvation shell in the PC/TEG solvent.

The structure of the solvation shell of Li becomes more complicated as we move to consider a 7 : 3 PC/TEG mixture. Both solvents are good ligands of the cation, PC via its dipole moment, TEG via its chelating nature. They indeed compete for Li^+^ coordination. The different solvating environment of Li^+^ can be seen in Figure 7. The most abundant configuration (21 %) is a 1 TFSI^−^, 3 PC coordination that is reminiscent of the situation previously found for the neat PC solution. Another likely coordination pattern (18 %) sees a tetracoordinated Li^+^ using two oxygen atoms from TFSI^−^ and PC and two oxygens from a single TEG molecule. There is also a residual population (11 %) where Li^+^ coordinates two TFSI anions. Both TEG and PC are in fact not able to prevent the formation of ionic complexes with two anions (see Figure 6).

The rather strong structuring of the solvents around the Li cation, results in a relatively low mobility of the ion itself. The computed data from our simulations of the 4 solutions are compared to the experimental data in Table 2. The computed densities are in excellent agreement with the experimental measurements. This again provides a further proof of the reliability of the developed FF in grasping the overall structural properties of the electrolyte. Much more difficult (even with polarization in the model potential) is however to predict reliably the delicate balance of intermolecular energies and dynamical processes that determine the local frictional properties of the fluid and, in turn, the Li^+^ mobility.

**Table 2:** Simulated (MD) and experimental (exp.) properties of the four 1 M LiTFSI electrolytes: densities (ρ), Li^+^ and TFSI^−^ diffusion coefficients (D±) and conductivities (σ).

	ρ (g ⋅ cm^−3^)		D_+_ (10^−11^ m^2^/s)	D_−_ (10^−11^ m^2^/s)	σ (mS ⋅ cm^−1^)	
Solvent	MD	exp.	MD	MD	MD	exp.
TMG	1.16	1.17^[a]^	2.49	3.24	2.2	1.5^[a]^
TEG	1.10	1.08^[a]^	1.13	1.48	1.0	1.5^[a]^
PC	1.35	1.32^[b]^	1.83	2.17	1.5	4.7^[c]^
PC : TEG	1.27	1.24^[c]^	1.42	2.01	1.3	3.5^[d]^

^[a]^ From ref. [13]; ^[b]^ from ref. [47]; ^[c]^ measured by us (details in section S1); ^[d]^ from ref. [20].

We have reported in Table 2 both the Li^+^ and TFSI^−^ diffusion coefficients as obtained by a linear fitting of their root mean square deviation (RMSD, see Section S3 for details). From these, an ideal value of the solution conductivity can be derived from the Nernst–Einstein formula where the ionic conductivity is simply proportional to the charge‐weighted sum of diffusion coefficients. The conductivity obtained from such method must be used with caution since it implies an ideal behavior of the solution, which is often not the case in a real, non‐ideal system. From the computed data in Table 2, the Li cation appears to have a higher mobility in TMG than in TEG. In particular, Li^+^ mobility in TMG is roughly twice the mobility in TEG and, in ideal conditions, this factor is simply transferred into the values of the computed conductivities. Experimental data, instead, point to the same conductivity in both solvents, thus highlighting a certain degree of non‐ideality of the real solutions probably due to the relatively high salt concentration and the ensuing dynamics of associated ionic couples. For both TEG and TMG the computed data are however not far from experimental values.

The discrepancy between our computed data and the experimental values widens for PC and PC/TEG mixture where we predict a value that is 3 times smaller than the experimental one. It is likely that in both these system, non‐ideality is more pronounced.

We note however that predicting the frictional/dynamical properties of a complex liquid system is a difficult task for molecular dynamics models even in ideal systems because of some intrinsic limits of the method, such as: the lack of a complete expansion of many body interactions; the presence of charge transfer effects (that can, for example, reduce the formal charge on Li^+^, hence local friction) and the absence of an electric field (that would account for reorientation dynamics of dipoles). We also stress that, given our choice of not carrying out additional (possibly empirically driven) re‐fitting of some FF parameters, these are already satisfactory results, considering that we used only ab‐initio data on isolated molecular components. To stress this point, we mention that, for comparison, a simulation of the PC:TEG electrolyte using a standard, non‐polarizable, OPLS FF the computed conductivity value is 0.4 mS cm^−1^, almost one order of magnitude below the experimental point.

## 4 Conclusions

In this paper, we have presented a new polarizable FF, based on the AMOEBA model for the simulation of three possible electrolytes in electrochemical devices. The FF (except for Li^+^) has been parametrized using high quality ab‐initio data computed on reference structures of the isolated molecular components of the electrolyte system. The force field turned out to reproduce the energy profiles along the dissociation pathway of Li^+^‐molecule complexes, and to match the cohesive energy of small neutral molecular clusters computed at the ab‐initio level. The FF was also validated in terms of prediction of bulk observables of the pure solvents (density, evaporation energy and conductivities) with decent accuracy.

The FF was used to characterize the structural and dynamical environment of the LiTFSI salt in the three chosen solvents. The structure of the first solvation shell of Li^+^, as well as the coexistence of different solvation patterns was described in detail and quantified using average configurational data. In essence, we have found that, despite its polar nature, PC is the less effective solvent in dissociating LiTFSI with the corresponding electrolyte containing a large prevalence (~80 %) of associated ionic couples (Figure 6‐d). Its binding to the Li^+^ cation is slightly weaker than the other solvents (Figure 2) and gives rise to a majority of tetra‐coordinated first shell structures with 3 PC molecules and one site occupied by TFSI (~48 % of the total LiTFSI).

In both TMG and TEG, owing to their bidentate ligand nature, the solvation shell of Li is penta‐coordinated and dominated by configurations with two bidentate solvent molecules with the fifth coordination site still used by TFSI. TMG, among all others, is the solvent that provides the largest number of dissociated Li^+^ ions (~30 %). In it, most of these Li^+^ (~25 % of the total Li) are surrounded by solvation shells that are essentially hexa‐coordinated by 3 TMG molecules.

In all solvents, when the LiTFSI is associated, it maintains a 1 : 1 stoichiometry, although in PC, a substantial fraction of the LiTFSI molecules (~20 %) appear to exist with a 1 : 2 ratio, i. e. as Li(TFSI)_2_. In TEG the Li(TFSI)_2_ fraction is significantly reduced, and it almost disappears in TMG.

The addition of TEG to PC does not significantly alter the local structure of Li^+^ with respect to PC alone, with the latter remaining the main solvating agent. Also, in the PC/TEG mixture the chance of having dissociated LiTFSI is slightly reduced (~16 %) with respect to PC alone, but the chance of having Li(TFSI)_2_ complexes is halved to only ~11 %.

## 5 Supporting Information

Experimental details; Simulation details; Force field parameters; RMSD plots of electrolytes solutions.

## Conflict of Interests

The authors declare no conflict of interest.

6

## Supporting information

As a service to our authors and readers, this journal provides supporting information supplied by the authors. Such materials are peer reviewed and may be re‐organized for online delivery, but are not copy‐edited or typeset. Technical support issues arising from supporting information (other than missing files) should be addressed to the authors.

Supporting Information

## Data Availability

The data that support the findings of this study are available from the corresponding author upon reasonable request.
