# Supplementary material for: A Polarizable Forcefields for Glyoxal Acetals as Electrolyte Components for Lithium‐Ion Batteries
Source: ChemistryOpen. 2024 Jul 31;13(11):e202400134. doi: 10.1002/open.202400134 (PMC11564869; doi:10.1002/open.202400134)

ChemistryOpen

Supporting Information

A Polarizable Forcefields for Glyoxal Acetals as Electrolyte Components for Lithium-Ion Batteries

Adriano Pierini, Vanessa Piacentini, Juan Luis Gómez-Urbano, Andrea Balducci, Sergio Brutti, and Enrico Bodo*

Supporting Information

A polarizable force fields for glyoxal acetals as electrolyte components for lithium-ion batteries

Adriano Pierini,[a] Vanessa Piacentini,[a] Juan Luis Gómez-Urbano,[b,c] Andrea Balducci,[b,c] Sergio Brutti,[a,d] and Enrico Bodo*[a]

[a] Dr. Adriano Pierini, Ms. Vanessa Piacentini, Prof. Sergio Brutti, Prof. Enrico Bodo
Department of Chemistry
University of Rome La Sapienza
P. Aldo Moro 5, 00185 Rome, Italy
E-mail: enrico.bodo@uniroma1.it

[b] Dr. Juan Luis Gómez-Urbano, Prof. Andrea Balducci
Institute for Technical Chemistry and Environmental Chemistry
Friedrich-Schiller University Jena.
Philosophenweg 7a, 07743 Jena, Germany

[c] Dr. Juan Luis Gómez-Urbano, Prof. Andrea Balducci
Center for Energy and Environmental Chemistry Jena (CEEC)
Friedrich-Schiller University Jena.
Philosophenweg 7a, 07743 Jena, Germany

[d] Prof. Sergio Brutti
Istituto dei Sistemi Complessi
Consiglio Nazionale delle Ricerche
P. Aldo Moro 5, 00185 Rome, Italy

S1. Experimental details for density and conductivity measurements

The solvent TEG (Weylchem) was initially pre-dried by an overpressure Schlenk filtration over aluminium oxide under vacuum until the water content was reduced from 2000 to 20 ppm as measured by Karl-Fischer titration (C20 Coulometric KF Titrator, METTLER TOLEDO). Following, TEG was dried over molecular sieves to further reduce its water content. LiTFSI (Solvionic, 99.9%) and PC (Sigma Aldrich, anhydrous 99.7%) were employed as received for the preparation of the electrolytes (1 M LiTFSI in PC and 1 M LiTFSI in PC/TEG 3:7 weight). The water content of the electrolytes was confirmed to be below 20 ppm.
The density value of 1M LiTFSI in PC/TEG (7:3) at 20 °C was registered by using an oscillating U-tube density meter DMA 4100 M (Anton Paar). The conductivity value of 1 M LiTFSI in PC has been measured using a ModuLab XM ECS potentiostat in a conductivity cell consisting of two parallel platinum electrodes. The determination was done at open circuit voltage by applying an alternating voltage with an amplitude of 5 mV, within a frequency range from 300 kHz to 1 Hz. The conductivity values were obtained after multiplying the as-obtained conductance by the known-cell constant that was previously measured using a 3 M KCl solution.

Table S1. Composition of the simulation cells

Electrolyte	N atoms	N mol. (solvent)	N mol. (salt)
TMG	15984	666	0
TEG	15984	444	0
PC	15990	1230	0
TMG + LiTFSI	12008	455	68
TEG + LiTFSI	11988	305	63
PC + LiTFSI	12004	820	84
PC/TEG (7:3) + LiTFSI	11998	522 PC + 111 TEG	76

S2. AMOEBA force field parameters in the Tinker format

TFSI.key

```
atom         1    1    C     "TFSI- C"                     6    12.011    4
atom         2    2    S     "TFSI- S"                    16    32.066    4
atom         3    3    N     "TFSI- N"                     7    14.007    2
atom         4    4    O     "TFSI- O"                     8    15.999    1
atom         5    5    F     "TFSI- F"                     9    18.998    1
vdw          1               3.8200     0.1010
vdw          2               3.9100     0.3850
vdw          3               3.7100     0.1100
vdw          4               3.4000     0.1000
vdw          5               3.2200     0.0610
bond         2    3          550.00     1.6100
bond         1    2          250.00     1.8400
bond         2    4          606.00     1.4700
bond         1    5          348.98     1.3500
angle        3    2    4      75.00     111.53
angle        1    2    3      75.00      92.48
angle        2    3    2      65.00     114.73
angle        2    1    5      60.00     108.24
angle        1    2    4      75.00     102.44
angle        4    2    4      80.00     121.78
angle        5    1    5      89.14     109.87
strbnd       3    2    4       0.00       0.00
strbnd       3    2    1       0.00       0.00
strbnd       2    3    2       0.00       0.00
strbnd       2    1    5       0.00       0.00
strbnd       5    1    5       5.71       5.71

torsion      5    1    2    3      1.500 0.0 1  -0.963 180.0 2   1.095 0.0 3
torsion      4    2    3    2     -9.798 0.0 1   5.169 180.0 2   6.577 0.0 3

# Note: This torsion from poltype produces only cisoid TFSI
torsion      1    2    3    2      2.230 0.0 1   5.532 180.0 2  -9.793 0.0 3
# Use this torsion for both cisoid and transoid TFSI
#torsion     1    2    3    2      2.230 0.0 1  -2.24  180.0 2  -0.09  0.0 3

torsion      5    1    2    4     -2.745 0.0 1   2.478 180.0 2  -0.220 0.0 3

multipole    1    2    3                0.59764
                                       -0.06173    0.00000   -0.28462
                                        0.40625
                                        0.00000    0.28817
                                       -0.14963    0.00000   -0.69442
multipole    2    1    3                1.29641
                                       -0.14077    0.00000    0.04086
                                       -0.06100
                                        0.00000    1.06990
                                        0.36904    0.00000   -1.00890
multipole    3   -2   -2               -0.65134
                                        0.00000    0.00000    0.35319
                                        0.30120
                                        0.00000   -0.69514
                                        0.00000    0.00000    0.39394
multipole    4    2    1               -0.69730
                                       -0.07142    0.00000    0.01967
                                       -0.25524
                                        0.00000   -0.28001
                                       -0.04944    0.00000    0.53525
multipole    5    1    0               -0.21888
                                        0.00000    0.00000    0.15334
                                       -0.23731
                                        0.00000   -0.23731
                                        0.00000    0.00000    0.47462
polarize     1          1.6196     0.3900      5
polarize     2          2.9941     0.3900      3    4
polarize     3          1.4437     0.3900      2
polarize     4          0.8588     0.3900      2
polarize     5          0.3481     0.3900      1
```

TMG.key

```
atom          6    6    C     "TMG CH"                      6    12.011    4
atom          7    7    C     "TMG CH3"                     6    12.011    4
atom          8    8    O     "TMG O"                       8    15.999    2
atom          9    9    H     "TMG HC"                      1     1.008    1
atom         10   10    H     "TMG H3C"                     1     1.008    1
vdw           6               3.8200     0.1010
vdw           7               3.8200     0.1010
vdw           8               3.3560     0.1188
vdw           9               2.8700     0.0240      0.910
vdw          10               2.9600     0.0240      0.920
bond          6    9          350.43     1.1000
bond          6    6          172.74     1.5200
bond          6    8          230.88     1.4100
bond          7    8          291.91     1.4200
bond          7   10          345.97     1.0900
angle         6    6    8      88.00     108.01
angle         6    6    9      41.78     110.08
angle         6    8    7      76.89     109.53
angle         6    6    9      41.78     110.08
angle         8    6    9      51.91     110.17
angle         8    6    8      91.51     111.27
angle         8    7   10      57.32     108.46
angle        10    7   10      72.81     109.35
strbnd        6    6    8       0.00       0.00
strbnd        9    6    6       5.71       5.71
strbnd        7    8    6       6.87       6.87
strbnd        6    6    9       5.71       5.71
strbnd        8    6    9       5.71       5.71
strbnd        8    6    8       5.71       5.71
strbnd       10    7    8       5.71       5.71
strbnd       10    7   10       5.71       5.71
torsion       6    6    8    7    -1.114 0.0 1   2.278 180.0 2   0.710 0.0 3
torsion       8    6    6    8     1.733 0.0 1  -0.985 180.0 2   0.937 0.0 3
torsion       8    6    8    7    -0.594 0.0 1  -0.281 180.0 2   0.389 0.0 3
torsion       8    6    8    7    -0.594 0.0 1  -0.281 180.0 2   0.389 0.0 3
torsion      10    7    8    6     0.000 0.0 1   0.000 180.0 2   0.108 0.0 3
torsion       8    6    6    9     0.000 0.0 1   0.000 180.0 2   0.108 0.0 3
torsion       9    6    6    9     0.000 0.0 1   0.000 180.0 2   0.299 0.0 3
torsion       9    6    8    7     0.000 0.0 1   0.000 180.0 2   0.597 0.0 3
multipole     6    9    6                0.32084
                                        -0.35206    0.00000   -0.31074
                                         0.06928
                                         0.00000    0.25605
                                         0.40042    0.00000   -0.32533
multipole     7  -10  -10  -10           0.05854
                                        -0.00462    0.00000   -0.32644
                                        -0.41020
                                         0.00000   -0.40219
                                        -0.01674    0.00000    0.81239
multipole     8    7    6               -0.34721
                                         0.14187    0.00000    0.26518
                                         0.19204
                                         0.00000   -0.72868
                                        -0.41611    0.00000    0.53664
multipole     9    6    6               -0.00402
                                         0.09218    0.00000   -0.08991
                                        -0.01269
                                         0.00000   -0.07122
                                        -0.00248    0.00000    0.08391
multipole    10    7    8                0.04342
                                         0.00382    0.00000   -0.09060
                                        -0.02262
                                         0.00000    0.05162
                                        -0.03761    0.00000   -0.02900
polarize      6          1.6196     0.3900      8    9
polarize      7          1.6196     0.3900      8   10
polarize      8          0.8122     0.3900      6    7
polarize      9          0.4803     0.3900      6
polarize     10          0.4803     0.3900      7
```

TEG.key

```
atom         11   11    C     "TEG CH"                      6    12.011    4
atom         12   12    H     "TEG HC"                      1     1.008    1
atom         13   13    H     "TEG H2C"                     1     1.008    1
atom         14   14    C     "TEG CH3"                     6    12.011    4
```

```
atom          15    15    O     "TEG O"                          8    15.999    2
atom          16    16    C     "TEG CH2"                        6    12.011    4
atom          17    17    H     "TEG H3C"                        1     1.008    1
vdw           11                3.6500     0.1010      0.000
vdw           12                2.8700     0.0240      0.910
vdw           13                2.8700     0.0240      0.910
vdw           14                3.8200     0.1010      0.000
vdw           15                3.4050     0.1100      0.000
vdw           16                3.8200     0.1010      0.000
vdw           17                2.9000     0.0220      0.900
bond          11    12          350.43     1.1100
bond          11    11          338.95     1.4867
bond          11    15          230.88     1.4000
bond          15    16          274.96     1.4300
bond          13    16          340.53     1.1000
bond          14    16          203.31     1.5100
bond          14    17          345.97     1.0900
angle         11    11    15    176.00     109.63
angle         11    11    12     83.56     109.27
angle         11    15    16     87.74     109.19
angle         11    11    15     67.75     109.63
angle         11    11    12     69.01     109.27
angle         11    15    16     87.74     109.71
angle         11    11    12     74.63     109.27
angle         12    11    15     51.91     109.54
angle         15    11    15     91.52     109.24
angle         13    16    15     54.88     110.08
angle         14    16    15     90.88     106.95
angle         16    14    17     50.88     110.21
angle         13    16    13     30.49     107.77
angle         13    16    14     47.06     110.99
angle         17    14    17     36.40     108.72
strbnd        11    11    15     20.45      20.45
strbnd        12    11    11     20.45      20.45
strbnd        16    15    11      6.87       6.87
strbnd        11    11    12     20.45      20.45
strbnd        15    11    12      5.71       5.71
strbnd        15    11    15      5.71       5.71
strbnd        13    16    15      5.71       5.71
strbnd        14    16    15      5.71       5.71
strbnd        17    14    16      5.71       5.71
strbnd        13    16    13      5.71       5.71
strbnd        14    16    13      5.71       5.71
strbnd        17    14    17      5.71       5.71
torsion       11    11    15    16       1.001 0.0 1   0.221 180.0 2   0.482 0.0 3
torsion       11    15    16    14      -1.204 0.0 1   0.325 180.0 2   0.651 0.0 3
torsion       15    11    11    15       0.129 0.0 1   0.208 180.0 2   0.179 0.0 3
torsion       15    11    15    16      -2.366 0.0 1   0.000 180.0 2   0.534 0.0 3
torsion       15    11    15    16      -2.366 0.0 1   0.000 180.0 2   0.534 0.0 3
torsion       11    15    16    13       0.000 0.0 1   0.000 180.0 2   0.108 0.0 3
torsion       12    11    15    16       0.000 0.0 1   0.000 180.0 2   0.108 0.0 3
torsion       17    14    16    15      -0.000 0.0 1  -0.000 180.0 2   0.000 0.0 3
torsion       12    11    11    15       0.000 0.0 1   0.000 180.0 2   0.108 0.0 3
torsion       17    14    16    13       0.000 0.0 1   0.000 180.0 2   0.415 0.0 3
torsion       12    11    11    12       0.000 0.0 1   0.000 180.0 2   0.299 0.0 3
multipole     11    11   -15   -15            0.27173
                                              0.42479     0.00000     0.09170
                                             -0.15496
                                              0.00000     0.17654
                                             -0.48648     0.00000    -0.02158
multipole     12    11    11                 -0.04059
                                             -0.01917     0.00000    -0.12114
                                             -0.07292
                                              0.00000     0.03421
                                             -0.00822     0.00000     0.03871
multipole     13    16    15                  0.01148
                                             -0.02844     0.00000    -0.09426
                                             -0.01284
                                              0.00000     0.04921
                                             -0.03714     0.00000    -0.03637
multipole     14    16    15                 -0.15465
                                             -0.00629     0.00000     0.23937
                                             -0.21696
                                              0.00000    -0.20157
                                              0.00308     0.00000     0.41853
multipole     15    11    16                 -0.27038
                                              0.46812     0.00000     0.15165
```

```
                                          0.23723
                                          0.00000    -0.78531
                                         -0.36860     0.00000     0.54808
multipole    16   15   14                 0.09720
                                          0.08220     0.00000     0.33539
                                         -0.25829
                                          0.00000    -0.33466
                                         -0.26667     0.00000     0.59295
multipole    17   14   16                 0.06310
                                          0.01614     0.00000    -0.05134
                                          0.00853
                                          0.00000    -0.01629
                                          0.01564     0.00000     0.00776
polarize     11          1.6200     0.3900     12   15
polarize     12          0.4800     0.3900     11
polarize     13          0.4800     0.3900     16
polarize     14          1.4150     0.3900     17
polarize     15          0.8120     0.3900     11   16
polarize     16          1.6200     0.3900     13   15
polarize     17          0.4800     0.3900     14
```

PC.key

```
atom         18   18    C     "PC CH"                     6    12.011    4
atom         19   19    C     "PC CH2"                    6    12.011    4
atom         20   20    C     "PC CH3"                    6    12.011    4
atom         21   21    C     "PC C=O"                    6    12.011    3
atom         22   22    O     "PC O-CH-"                  8    15.999    2
atom         23   23    O     "PC O-CH2-"                 8    15.999    2
atom         24   24    H     "PC H2C"                    1     1.008    1
atom         25   25    H     "PC H3C"                    1     1.008    1
atom         26   26    H     "PC HC"                     1     1.008    1
atom         27   27    O     "PC O="                     8    15.999    1
vdw          18               3.8200     0.1010
vdw          19               3.8200     0.1010
vdw          20               3.8200     0.1010
vdw          21               3.8000     0.0910
vdw          22               3.3560     0.1188
vdw          23               3.4050     0.1100
vdw          24               2.9100     0.0330      0.900
vdw          25               2.9600     0.0240      0.920
vdw          26               2.9600     0.0240      0.920
vdw          27               3.4000     0.1000
bond         21   27          292.72     1.2000
bond         21   23          292.72     1.3700
bond         21   22          292.72     1.3700
bond         18   23          249.68     1.4500
bond         19   22          326.04     1.4400
bond         19   24          352.50     1.0900
bond         18   19          221.54     1.5200
bond         18   26          352.50     1.1000
bond         18   20          280.35     1.5100
bond         20   25          345.97     1.0900
angle        23   21   27      54.46     125.10
angle        22   21   27      54.46     124.83
angle        18   23   21      91.36     108.83
angle        19   22   21      84.52     110.75
angle        22   21   23      54.46     111.66
angle        19   18   23      85.42     105.09
angle        23   18   26      57.38     111.16
angle        20   18   23      82.20     109.56
angle        22   19   24      55.07     110.04
angle        18   19   22      85.42     105.50
angle        19   18   26      50.58     110.56
angle        19   18   20      97.74     115.71
angle        24   19   24      35.47     112.90
angle        18   19   24      50.58     111.52
angle        18   20   25      47.42     110.17
angle        20   18   26      44.31     107.84
angle        25   20   25      36.40     108.76
strbnd       23   21   27       7.62       7.62
strbnd       22   21   27       7.62       7.62
strbnd       18   23   21      21.33     -21.33
strbnd       19   22   21      21.33     -21.33
strbnd       22   21   23       7.62       7.62
strbnd       19   18   23      27.93      27.93
```

```
strbnd        26   18   23        5.71       5.71
strbnd        20   18   23        5.71       5.71
strbnd        24   19   22        5.71       5.71
strbnd        18   19   22       27.93      27.93
strbnd        26   18   19       27.93      27.93
strbnd        20   18   19        5.71       5.71
strbnd        24   19   24       27.93      27.93
strbnd        18   19   24       27.93      27.93
strbnd        25   20   18        5.71       5.71
strbnd        20   18   26        5.71       5.71
strbnd        25   20   25        5.71       5.71
opbend        27   21    0    0              36.30
opbend        23   21    0    0              36.30
opbend        22   21    0    0              36.30
torsion       27   21   23   18       0.000 0.0 1   0.000 180.0 2    0.299 0.0 3
torsion       27   21   22   19       0.000 0.0 1   0.000 180.0 2    0.299 0.0 3
torsion       18   19   22   21       0.854 0.0 1  -0.374 180.0 2    0.108 0.0 3
torsion       23   21   22   19       0.000 0.0 1   0.000 180.0 2    0.299 0.0 3
torsion       22   21   23   18       0.000 0.0 1   0.000 180.0 2    0.299 0.0 3
torsion       24   19   22   21       0.000 0.0 1   0.000 180.0 2    0.108 0.0 3
torsion       20   18   19   22       0.854 0.0 1  -0.374 180.0 2    0.108 0.0 3
torsion       20   18   19   24       0.000 0.0 1   0.000 180.0 2    0.108 0.0 3
torsion       19   18   20   25       0.000 0.0 1   0.000 180.0 2    0.108 0.0 3
torsion       23   18   19   24       0.000 0.0 1   0.000 180.0 2    0.108 0.0 3
torsion       23   18   20   25       0.000 0.0 1   0.000 180.0 2    0.108 0.0 3
torsion       26   18   20   25       0.000 0.0 1   0.000 180.0 2    0.238 0.0 3
torsion       26   18   23   21       0.000 0.0 1   0.000 180.0 2    0.597 0.0 3
torsion       20   18   23   21       4.389 0.0 1  -1.168 180.0 2   -4.389 0.0 3
torsion       23   18   19   22      -0.003 0.0 1  -0.360 180.0 2    0.684 0.0 3
torsion       26   18   19   22       0.000 0.0 1   0.000 180.0 2    0.108 0.0 3
torsion       26   18   19   24       9.655 0.0 1  -0.271 180.0 2   15.312 0.0 3
torsion       19   18   23   21       0.883 0.0 1  -0.780 180.0 2    0.520 0.0 3
multipole     18   20   26                 0.03966
                                          -0.17224    0.00000   -0.13830
                                          -0.47447
                                           0.00000    0.56758
                                           0.22784    0.00000   -0.09311
multipole     19   18   22                 0.04499
                                           0.24165    0.00000    0.21525
                                           0.54015
                                           0.00000   -0.58567
                                          -0.52258    0.00000    0.04552
multipole     20   18   26                -0.19071
                                           0.01525    0.00000    0.24695
                                          -0.20251
                                           0.00000   -0.13070
                                           0.02295    0.00000    0.33321
multipole     21   22   23                 0.95791
                                           0.16583    0.00000    0.08978
                                           0.14996
                                           0.00000   -0.19248
                                          -0.06396    0.00000    0.04252
multipole     22   19   21                -0.36557
                                           0.10375    0.00000    0.27204
                                          -0.04960
                                           0.00000   -0.48759
                                          -0.10743    0.00000    0.53719
multipole     23   18   21                -0.32567
                                           0.12915    0.00000    0.36522
                                          -0.20635
                                           0.00000   -0.48502
                                          -0.02394    0.00000    0.69137
multipole     24   19   18                 0.07305
                                           0.01785    0.00000   -0.11692
                                           0.08977
                                           0.00000    0.06163
                                           0.02448    0.00000   -0.15140
multipole     25   20   18                 0.08102
                                           0.00221    0.00000   -0.02701
                                          -0.02602
                                           0.00000   -0.02453
                                           0.00502    0.00000    0.05055
multipole     26   18   20                 0.08267
                                           0.05367    0.00000   -0.00021
                                           0.02971
                                           0.00000   -0.05944
                                           0.04960    0.00000    0.02973
```

```
multipole    27   21   22              -0.63244
                                        0.01760    0.00000   -0.09309
                                       -0.29347
                                        0.00000    0.13674
                                        0.04141    0.00000    0.15673
polarize     18          1.6196     0.3900     26
polarize     19          1.6196     0.3900     24
polarize     20          1.4150     0.3900     25
polarize     21          2.0645     0.3900     22   23   27
polarize     22          0.8323     0.3900     21
polarize     23          0.8323     0.3900     21
polarize     24          0.4803     0.3900     19
polarize     25          0.4803     0.3900     20
polarize     26          0.4803     0.3900     18
polarize     27          0.9138     0.3900     21
```

AMOEBA09.prm

```
forcefield              AMOEBA-2009
bond-cubic              -2.55
bond-quartic            3.793125
angle-cubic             -0.014
angle-quartic           0.000056
angle-pentic            -0.0000007
angle-sextic            0.000000022
opbendtype              ALLINGER
opbend-cubic            -0.014
opbend-quartic          0.000056
opbend-pentic           -0.0000007
opbend-sextic           0.000000022
torsionunit             0.5
vdwtype                 BUFFERED-14-7
radiusrule              CUBIC-MEAN
radiustype              R-MIN
radiussize              DIAMETER
epsilonrule             HHG
dielectric              1.0
polarization            MUTUAL
vdw-12-scale            0.0
vdw-13-scale            0.0
vdw-14-scale            1.0
vdw-15-scale            1.0
mpole-12-scale          0.0
mpole-13-scale          0.0
mpole-14-scale          0.4
mpole-15-scale          0.8
polar-12-scale          0.0
polar-13-scale          0.0
polar-14-scale          1.0
polar-15-scale          1.0
polar-12-intra          0.0
polar-13-intra          0.0
polar-14-intra          0.5
polar-15-intra          1.0
direct-11-scale         0.0
direct-12-scale         1.0
direct-13-scale         1.0
direct-14-scale         1.0
mutual-11-scale         1.0
mutual-12-scale         1.0
mutual-13-scale         1.0
mutual-14-scale         1.0
```

S3. MSD fitting

Ionic self-diffusion coefficients were calculated from molecular dynamics simulations according to the Einstein relation:

$$D_\alpha = \frac{1}{6}\frac{d}{dt}\left\langle \frac{1}{N}\sum_{i=1}^{N} |r_{\alpha,i}(t) - r_{\alpha,i}(0)|^2 \right\rangle$$

The following plots show the MSDs (dotted lines) and the linear fits (solid lines) from which the numerical values of self-diffusion coefficients were calculated.

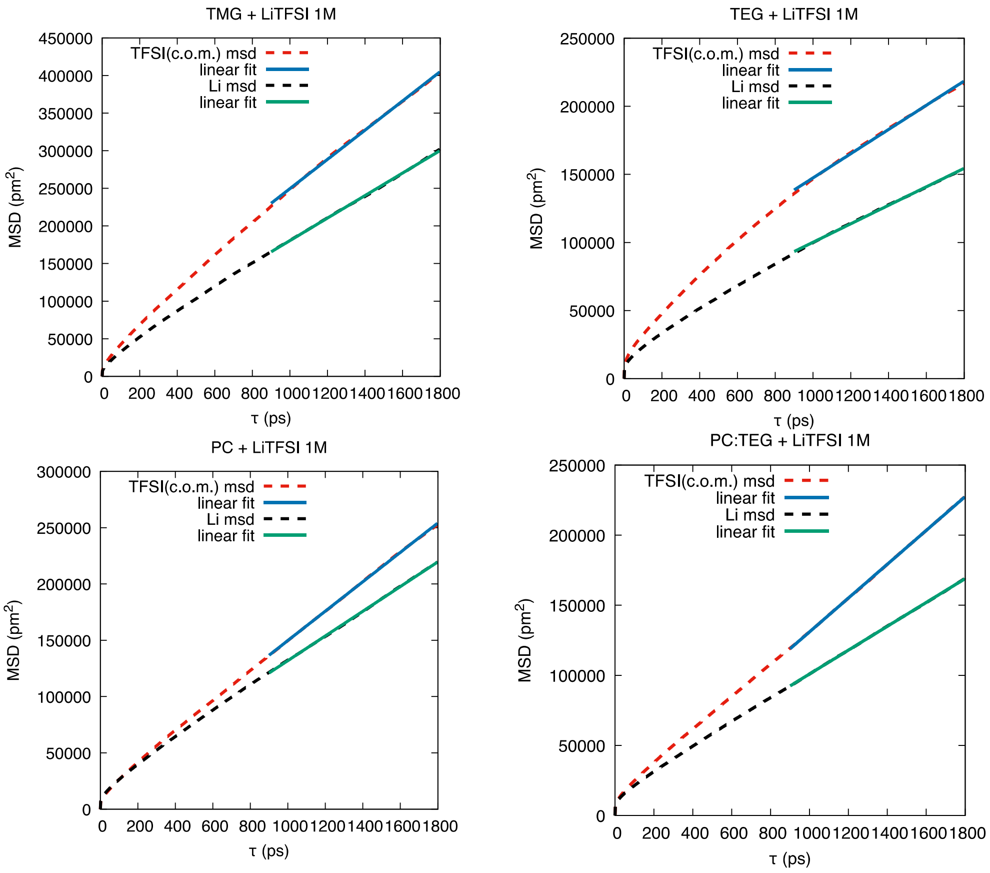

Supplement: Supplementary file 1 — Supporting Information [file OPEN-13-e202400134-s001.pdf]
